# Whole pulmonary assessment 1 year after paediatric acute respiratory distress syndrome: prospective multicentre study

**DOI:** 10.1186/s13613-022-01050-4

**Published:** 2022-08-20

**Authors:** Véronique Nève, Ahmed Sadik, Laurent Petyt, Stéphane Dauger, Ahmed Kheniche, André Denjean, Pierre-Louis Léger, François Chalard, Michèle Boulé, Etienne Javouhey, Philippe Reix, Isabelle Canterino, Valérie Deken, Régis Matran, Stéphane Leteurtre, Francis Leclerc

**Affiliations:** 1 Pulmonary Function Testing Department, CHU Lille, 2 avenue Oscar Lambret, 59000 Lille, France; 2 Univ. Lille, ULR 4483, IMPECS, 59000 Lille, France; 3 Institut Pasteur de Lille, 59000 Lille, France; 4 Réanimation pédiatrique, CHU Lille, 59000 Lille, France; 5 Imaging Department, University Hospital, 59000 Lille, France; 6 Pediatric Intensive Care Unit, Assistance Publique, Hôpitaux de Paris, Robert Debré University Hospital, Université de Paris, Paris, France; 7 Imaging Department, Assistance Publique, Hôpitaux de Paris, Robert Debré University Hospital, Paris, France; 8 Pulmonary Function Testing Department, Assistance Publique, Hôpitaux de Paris, Robert Debré University Hospital, Université de Paris, Paris, France; 9 Pediatric and Neonatal Intensive Care Unit, Assistance Publique, Hôpitaux de Paris, Armand-Trousseau Hospital, Sorbonne University, Paris, France; 10 Imaging Department, Assistance Publique, Hôpitaux de Paris, Armand-Trousseau Hospital, Sorbonne University, Paris, France; 11 Pulmonary Function Testing Department, Assistance Publique, Hôpitaux de Paris, Armand- Trousseau University Hospital, Sorbonne University, Paris, France; 12 Pediatric Intensive Care Unit, Hôpital Femme Mère Enfant, Hospices Civils de Lyon, Université Lyon1, Lyon, France; 13 Pediatric Pulmonology Department, Hôpital Femme Mère Enfant, Hospices Civils de Lyon, Université Lyon1, Lyon, France; 14 Imaging Department, Hôpital Femme Mère Enfant, Hospices Civils de Lyon, Université Lyon1, Lyon, France; 15 Service de Biostatistique et Data Management, CHU Lille, 59000 Lille, France; 16 Évaluation des technologies de santé et des pratiques médicales, Univ. Lille, ULR 2694, METRICS, 59000 Lille, France

**Keywords:** ARDS, Child, Long-term outcomes, Computed tomography, Pulmonary function

## Abstract

**Background:**

Long-term pulmonary sequelae, including 1-year thoracic computed tomography (CT) sequelae of paediatric acute respiratory distress syndrome (ARDS) remain unknown. The purpose of the study was to determine pulmonary abnormalities in child survivors of pulmonary (p-ARDS) and extra-pulmonary ARDS (ep-ARDS) 1 year after paediatric intensive care unit discharge (PICUD).

**Methods:**

Prospective multicentre study in four paediatric academic centres between 2005 and 2014. Patients with ARDS were assessed 1 year after PICUD with respiratory symptom questionnaire, thoracic CT and pulmonary function tests (PFT).

**Results:**

39 patients (31 p-ARDS) aged 1.1–16.2 years were assessed. Respiratory symptoms at rest or exercise and/or respiratory maintenance treatment were reported in 23 (74%) of children with p-ARDS but in 1 (13%) of those with ep-ARDS. Thoracic CT abnormalities were observed in 18 (60%) of children with p-ARDS and 4 (50%) of those with ep-ARDS. Diffuse and more important CT abnormalities, such as ground glass opacities or mosaic perfusion patterns, were observed in 5 (13%) of children, all with p-ARDS. PFT abnormalities were observed in 30 (86%) of patients: lung hyperinflation and/or obstructive pattern in 12 (34%) children, restrictive abnormalities in 6 (50%), mild decrease in diffusing capacity in 2 (38%) and 6-min walking distance decrease in 11 (73%). Important PFT abnormalities were observed in 7 (20%) children, all with p-ARDS. Increasing driving pressure (max plateau pressure—max positive end-expiratory pressure) was correlated with increasing CT-scan abnormalities and increasing functional residual capacity (more hyperinflation) (*p* < 0.005).

**Conclusions:**

Children surviving ARDS requiring mechanical ventilation present frequent respiratory symptoms, significant CT-scan and PFT abnormalities 1 year after PICUD. This highlights the need for a systematic pulmonary assessment of these children.

*Trial registration* The study was registered on Clinical Trials.gov PRS (ID NCT01435889)

**Supplementary Information:**

The online version contains supplementary material available at 10.1186/s13613-022-01050-4.

## Background

Although outcomes for paediatric acute respiratory distress syndrome (ARDS) have improved over the last decade, mortality remains significant [1]. Persistent abnormal respiratory symptoms are still described 3 months after discharge in a significant proportion of paediatric ARDS survivors [2]. In survivors, the long-term consequences of paediatric ARDS remain largely unknown as existing studies are generally small and their results are probably outdated in the current era of lung-protective ventilation [3]. To our knowledge, there is no study describing the 1-year-thoracic computed tomography (CT) abnormalities persisting in children who survived ARDS. Such a study should differentiate ARDS resulting from pulmonary (p-ARDS) and extra-pulmonary causes (ep-ARDS) as more severe abnormalities, including ground-glass opacity, were described on thin-section of CT of adults survivors of p-ARDS [4]. The Paediatric Acute Lung Injury Consensus Conference (PALICC) Group recommended screening for pulmonary function abnormalities (respiratory symptom questionnaire, pulse oximetry and spirometry) in children with sufficient developmental age and capabilities within the first year after discharge for all ARDS who have undergone invasive mechanical ventilation [1]. The aim of the study was to screen for pulmonary abnormalities (respiratory symptoms, abnormalities in pulmonary function tests (PFT) and, for the first time, abnormalities in thoracic CT scans) 1 year after discharge from paediatric intensive care unit (PICU) in children surviving p-ARDS and ep-ARDS, separately.

## Methods

### Study design and data collection

Six university-affiliated paediatric intensive care units (PICU) in France and Belgium, all members of the Groupe Francophone de Réanimation et Urgences Pédiatriques (gfrup.sfpediatrie.com) and of the Réseau Mère-Enfant de la Francophonie (rmefrancophonie.org), accepted to participate in this multicentre prospective study, but only 4 PICU included patients.

The study and its database were declared safe and approved by the institutional ethics committee of Lille University Hospital on October 10th 2004 and by the French authorities (Commission Nationale de l’Informatique et des Libertés). It was supported, in part, by Grant from the French Ministry of Health (Programme Hospitalier de Recherche Clinique Régional: 2005R/1906) and was registered on Clinical Trials.gov PRS (ID NCT01435889). The scheduled beginning of the study was June 2006 (as indicated in Clinical Trials), but first enrolment was on October 26th 2006. Date of 1-year assessment of the first included patient was December 27th 2006 and date of last patient’s respiratory assessment performed 1-year after PICU discharge (PICUD) was October 23rd 2014. Pulmonary assessment (respiratory symptom questionnaire (see Additional file 1), thoracic CT assessment of abnormalities of lung parenchyma and PFT) was performed 1 year after PICUD, in outpatients’ clinic, in each centre and pooled.

### Inclusion and exclusion criteria

Included patients had been hospitalized in PICU for severe lung injury characterized by acute onset (within less than 48 h), had a PaO_2_/FiO_2_ ratio ≤ 200 (for a least 6 h) while receiving invasive mechanical ventilation with a positive end-expiratory pressure (PEEP) of at least 5 cmH_2_O, had diffuse bilateral pulmonary infiltrates on chest radiography, no clinical or echography signs of left-heart failure, and an identifiable risk factor for ARDS, according to the American European Consensus Conference (AECC) definition [5]. AECC parameters, apart from the delay between the known lung injury and ARDS development, are in accordance with the PALICC definition [6]. Protocols of mechanical ventilation were defined by each PICU team according to current literature recommendations. Extracorporeal Life Support were available in all centres. The 1-year respiratory assessment could be proposed to the child’s parents during the PICU stay of their child or during the year following the PICUD, and consent could be signed during the PICU stay until the day of the 1-year assessment. Patients with evidence of pre-existing chronic lung disease, with clinical expression such as asthma, with neurologic or neuromuscular disorder, severe kidney failure, liver disease, cardiac or endocrine disease or psychiatric disorder and children under legal protection or unable to cooperate, at least passively, to the tests were excluded. No child was O_2_ dependent, neither on non-invasive ventilation. The patients had to be 3–16-year-old at the time of the 1-year assessment (original protocol).

### Thoracic CT scan

CT was performed with 1.0 mm collimation (5 mm intervals) with patients in the supine position. CT was performed at end-inspiration. Reconstruction algorithms were appropriate for viewing the lung parenchyma. Three anatomically comparable levels were analysed by one senior radiologist in each centre: level 1, which was the aortic arch; level 2, which was between the carina and pulmonary venous confluence; and level 3, which was 1 cm above the right hemidiaphragm. Each level was divided into four quadrants: right anterior, right posterior, left anterior, and left posterior. For each quadrant, observers determined the extent of ground glass opacities, parenchymal consolidations, septal reticulation, honey combing, centrilobular cysts, paraseptal cysts, fissure thickening and distortions, bronchiectasis, mosaic perfusion. The extent of involvement for each pattern was assigned a numerical score, where 0 = no involvement; 1 ≤ 25% involvement; 2 = 25–50% involvement; 3 > 50% involvement. For fissure thickening and distortions, the numerical scores were: 0 = no involvement and 1 = involvement. For each pattern, the score (ranging from 0 to 36, except for the fissure thickening and distortion pattern ranging from 0 to 12) was calculated by summing all quadrant values for each of slice, and adding these values together. Higher scores indicated more involvement by a given pattern.

### PFT

Patient underwent PFT appropriate for age and ability to cooperate.

In children less than 7 years at PFT, tests requiring no active cooperation were used for 2.5–6-year-old children or were performed during sleep, for children less than 2.5 years of age. Assessment included measurement of functional residual capacity by a helium dilution technique (FRC) to detect hyperinflation (FRC > 120% of predicted), lung mechanics, including lung resistance (R_L_, using the oesophageal-balloon technique) or airway resistance (R_int_, interruption method) to evaluate proximal obstruction, dynamic lung compliance (C_Ldyn_, oesophageal-balloon technique) to determine abnormalities in distal bronchi and/or interstitium, and sniff nasal inspiratory pressure measurement (SNIP) to evaluate inspiratory muscle strength. In addition, resting pulse oximetry ± arterialized capillary blood gases (when pulse oximetry was < 96% or when lung mechanics were evaluated during sleep in children younger than 2.5 years of age) ± a 6-min-walk test (6MWT) were performed (all tests depending on the age and cooperation of the child).

In children of at least 7 years of age, FRC and total lung capacity (TLC) were measured in order to detect either a true restrictive ventilatory defect (TLC < 80% of predicted) or an hyperinflation (TLC or FRC > 120% of predicted), forced spirometry was performed to detect either an obstructive ventilatory defect (forced expiratory volume in 1 s (FEV_1)_/forced vital capacity (FVC) ratio < lower limit of normal (LLN) or a pattern considered suggestive of a restrictive ventilatory defect (an isolated decrease in FVC < LLN together with a FEV_1_/FVC ratio ≥ LLN), lung transfer factor for CO (T_LCO_, single breath method) was measured to detect interstitial abnormalities, and SNIP measurement to evaluate inspiratory muscle strength. In addition, resting pulse oximetry ± arterialized capillary blood gases (when pulse oximetry was < 96%) and a 6MWT were performed.

All functional variables were expressed as the percentage of predicted mean values for height, except those of forced spirometry, T_LCO_, 6MWT and SNIP which were expressed as *z*-scores. For forced spirometry and T_LCO_, *z*-scores derived from the Global Lung Initiative (GLI) prediction equation [7, 8] were calculated using the software provided by the GLI group [9], and the LLN was defined as the 5th centile (i.e., − 1.645 *z*-score) as recommended by the American Thoracic and European Respiratory Societies [7, 10, 11].

### Statistical analyses

Results were expressed as medians (interquartile ranges (IQR)) for quantitative variables, and frequencies and percentages for categorical variables. Bivariate comparisons between most children with p-ARDS and in a minority of those with ep-ARDS were performed using Mann–Whitney U test for quantitative variables and Fisher’s exact test for categorical variables. The correlation between maximal expiratory tidal volume (VTE) and minimum respiratory system compliance (Crs) was evaluated using the Spearman’s correlation coefficient. The evaluations of the relationship between the existence of respiratory symptoms and CT abnormalities of lung parenchyma or between the existence of respiratory symptoms and pulmonary function abnormalities were studied with Chi-square’s test or Fisher’s exact test. A correlation matrix (spearman correlation) of physiologic parameters/ventilator settings, CT scan, and PFTs was created. Statistical testing was done at the two-tailed α level of 0.05. No corrections for multiple testing were done regarding the exploratory nature of the present study and results should be interpreted with caution and as hypothesis-generating. Data were analysed using the SAS software, version 9.4 (SAS Institute, Cary, NC).

## Results

**Fig. 1:**
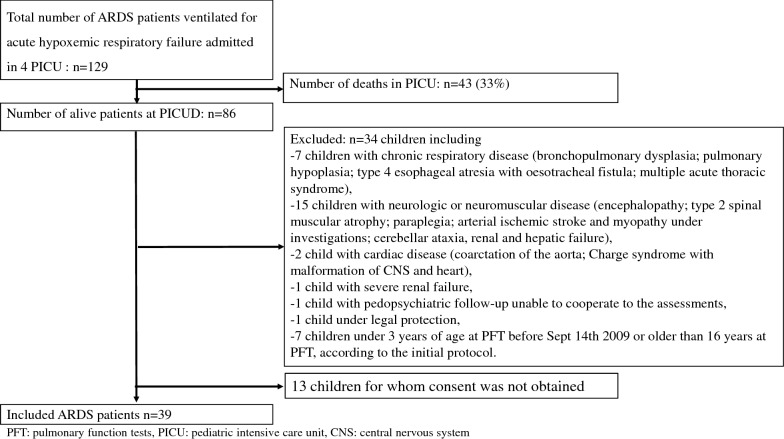
Flowchart

This study was initiated in five university-affiliated PICU. Their recruitment potential had been assessed on 2 years (2002 and 2003): 53 children hospitalized for an ARDS and surviving at the end the hospitalization were corresponding to the study criteria. Taking into account 10% of refusal, 45 children 3–16-year-old at the 1-year assessment were expected to be included. However, from September 2005 to September 2009, only 9 patients had had their 1-year assessment, therefore we decided to include younger children (1–16-year-old at the 1-year assessment from September 14th 2009). In July 2011, only 22 patients had been included in 2 centres. Thus a French Centre was added (i.e., total of 6 university-affiliated PICU were from that time participating to the study), duration of enrolment was increased from 5 to 8 years. Finally, only 4 PICU included patients for a total of 39 children among 129 children ventilated for ARDS in these 4 PICU (see the flowchart in Fig. 1).

Lille CHU included 16 patients (admitted from July 26th 2006 to March 29th 2013). Robert Debré University Hospital, Paris, included 13 patients (admitted from September 27th 2005 to December 16th 2011). Armand-Trousseau Hospital, Paris, included 6 patients (admitted from May 21st 2012 to November 14th 2013). Hospices Civils de Lyon Hospital included 4 patients (admitted from November 30th 2012 to March 30th 2013). Of the 39 included patients (Table 1), 67% were boys.

**Table 1:** Aetiology of ARDS of the 39 children

Pulmonary ARDS (p-ARDS) *n* = 31 (79.5%)
Diffuse pulmonary infection: bacterial (*n* = 7), viral (*n* = 8) or both (*n* = 1)
Aspiration (*n* = 2)
Toxic fume inhalation (*n* = 3)
Near drowning (*n* = 1)
Pneumocystic pneumonia or opportunistic infection (*n* = 4)
Acute chest syndrome of sickle cell disease (*n* = 1)
Foreign body inhalation (*n* = 1)
Alveolar haemorrhage (*n* = 2)
Ethylenglycol inhalation (*n* = 1)
Extra-pulmonary ARDS (ep-ARDS) *n* = 8 (20.5%)
Burns (*n* = 2)
Septic shock (*n* = 5)
Acute necrotizing pancreatitis and leukaemia (*n* = 1)

Characteristics of the patients during their PICU stay, according to the type of ARDS, are described in Table 2. Median maximal VTE, reported in 37 children, was 7.8 ml/kg (IQR: 6.4–11.1); 23 /37 (62%) were ventilated within the 5–8 ml/kg range, 14/37 (38%) within the 3 to 6 ml/kg range, as previously recommended [12], and 14/37 children (38%) within the 9 to 15 ml/kg range. Maximal VTE tended to decrease with minimum Crs/kg (r spearman = 0.392, *p* = 0.026). Maximal inspiratory pressure (PImax) was ≤ 40 cmH_2_O in 42% of patients in our study. Patients with p-ARDS had a shorter PICU stay and were ventilated with lower max PEEP than those with ep-ARDS.

**Table 2:** Characteristics of the 39 children during their PICU stay by type of ARDS

	p-ARDS *n* = 31	ep-ARDS *n* = 8	*p* value
Age (years)	1.7 (0.3–5.9)	5.80 (0.7–8.8)	0.32
Weight (kg)	11.4 (5.5–22)	21 (8.2–30)	0.31
PIM2 (%)	3.8 (1.5–10.4)	5.9 (4–13.1)	0.28
Duration of PICU stay (days)	14 (10–26)	29 (18–38)	0.038
Duration of invasive mechanical ventilation (days)	10 (7–16)	14 (10–31.5)	0.08
Duration of non-invasive mechanical ventilation (days)	2 (0–4)	0 (0–1.5)	0.12
Duration of O_2_ dependence (days)	11 (5–21)	15.5 (8–28.5)	0.42
Length of hospital stay (days)	19.5 (11.5–38.5)	33.5 (27–42)	0.054
Max VTE (ml/kg)	7.8 (6.4–11.1)	7.8 (6.1–10.6)	0.94
Max PEEP (cmH_2_O)	10 (8–12)	13 (11–14.9)	0.046
Max PImax (cmH_2_O)	44 (34–45)	46 (37–58	0.41
Max Pplat (cmH_2_O)	28 (24–38)	32 (26–45)	0.21
Duration of ventilation with inverse I/E ratio (days)	0 (0–0)	0 (0–1)	0.40
Max FiO_2*_	100 (100–100)	100 (92.5–100)	0.091
Worse PaO_2_/FiO_2_ ratio*	58 (49–69)	46.5 (40–71.3)	0.20
Days with FiO_2_ > 60%	5 (3–10)	5.5 (3–9.5)	0.73
Max resp PELOD	10 (10–10)	10 (1–10)	0.10
Max total PELOD	21 (20–22)	20 (12–21)	0.36
Max Lung Injury Score	3.3 (3.3–3.8)	3.50 (3.0–3.8)	0.81
Max Oxygenation Index	31.4 (23.7–40)	32.6 (22.6–57.1)	0.66
Max Ventilation Index	630 (480–760)	897 (644–1088)	0.14
General corticosteroid administration	25 (80.7%)	7 (87.5%)	1
Hydrocortisone total dose/body weight (mg/kg)	67.6 (27.6–96)	38.7 (14.8 to 117.4)	0.78
Hydrocortisone bolus	7 (29.2%)	3 (42.8%)	0.65
HFO	15 (48.4%)	1 (12.5%)	0.11
NO	14 (46.7%)	3 (37.5%)	0.71
ECLS	6 (20.0%)	0	0.31
Neuromuscular blocking agents	24 (77.4%)	5 (62.5%)	0.40
Surfactant administration	1 (3.2%)	0	NA

*Crs* respiratory system compliance, *ECLS* extracorporeal life support, *ep-ARDS* extra-pulmonary, *HFO* high-frequency oscillation, *I/E ratio* inspiratory/expiratory ratio, *p-ARDS* pulmonary ARDS, *PEEP* positive end-expiratory pressure, *PELOD score* Paediatric Logistic Organ Dysfunction Score, *PICU* paediatric intensive care unit, *PIMax* maximal inspiratory pressure, *PIM2* paediatric index of mortality score 2, *Pplat* plateau pressure, *NA* not applicable, *NO* inhaled nitric oxide, *VTE* expiratory tidal volume

Results are medians (interquartile ranges) for quantitative variables, and frequencies and percentages for categorical variables. *For NO: missing data in 1 child. *Worst PaO_2_/FiO_2_ ratio and max FiO_2_ during the PICU stay

### Respiratory symptoms and maintenance treatment

The medical history was reported by the patient and/or his/her parents the day of the 1-year respiratory assessment. During the year following PICUD, respiratory symptoms at rest or exercise and/or requirement for respiratory maintenance treatment (*p* = 0.0026, Table 3) were more likely in p-ARDS than in ep-ARDS group: in particular, wheezing episodes and requirement for maintenance treatment including inhaled rapid-acting beta 2-agonists or inhaled glucocorticosteroids (*p* < 0.05, Table 3) were more likely in p-ARDS than in ep-ARDS group.

**Table 3:** Symptoms and treatments in the year after PICU discharge in 39 children by ARDS type

	p-ARDS *n* = 31	ep-ARDS *n* = 8	*p* value
Time after discharge (year)	1.06 (1–1.11)	1.05 (0.94–1.11)	0.75
Respiratory symptoms at rest, exercise, maintenance treatment	23 (74.2%)	1 (12.5%)	0.0026
Chronic cough	5 (16.1%)	0	0.56
Wheezing episodes	16 (51.6%)	0	0.012
A least 3 wheezing episodes	10 (32.3%)	0	NA
Lower respiratory tract infection	11 (35.5%)	0	0.078
A least 3 episodes	3 (9.7%)	0	NA
Maintenance respiratory treatment	19 (63.3%)	0	0.003
Inhaled glucocorticosteroids	16 (51.6%)	0	0.012
Inhaled long-acting β2-agonists	7 (22.6%)	0	0.31
Inhaled rapid-acting β2-agonists	14 (45.2%)	0	0.034
Inhaled rapid-acting atropinic	1 (3.2%)	0	NA
Leukotriene modifier	2 (6.5%)	0	NA
Oxygen therapy	1 (3.2%)	0	NA
Course of systemic glucocorticosteroids in the year	4 (12.9%)	0	NA
Antibiotics	9 (29.0%)	0	0.16

*ep-ARDS* extra-pulmonary ARDS, *p-ARDS* pulmonary ARDS. Results are medians (interquartile ranges) for quantitative variables, and frequencies and percentages for categorical variables

### Thoracic CT scan

Thoracic CT scans were obtained in 38/39 children 1 year after PICUD**.** CT-scan abnormalities (60% versus 50%, *p* = 0.70, Table 4), and their anterior distribution were observed in a similar proportion of p-ARDS and ep-ARDS groups while a posterior distribution was more frequent in p-ARDS than ep-ARDS group (53% versus 0%, *p* = 0.012). Most patients showed moderate abnormalities as described by medians and IQRs (Table 4). However, p-ARDS had more important CT-scan abnormalities in the posterior quadrants (Table 4).

**Table 4:** CT-scan abnormalities observed in 38 children by type of ARDS

	Qualitative analysis			Sum of quotations	
	p-ARDS *n* = 30	ep-ARDS *n* = 8	*p* value	p-ARDS *n* = 30	ep-ARDS *n* = 8
CT-scan abnormalities	18 (60%)	4 (50%)	0.70	1 (0–8), *55*	0.5 (0–5.5), *9*
Anterior distribution	13 (43.3%)	4 (50%)	1	0 (0–5), *25*	0.5 (0–5.5), *9*
Posterior distribution	16 (53.3%)	0	0.012	1 (0–7), *30*	0 (0–0), *0*
Ground glass opacities	7 (23.3%)	1 (12.5%)	0.66	0 (0–0), *31*	0 (0–0), *4*
Parenchymal consolidations	6 (20%)	1 (12.5%)	1	0 (0–0), *9*	0 (0–0), *1*
Septal reticulation	4 (13.3%)	1 (12.5%)	1	0 (0–0), *8*	0 (0–0), *3*
Honey combing	1 (3.3%)	1 (12.5%)	NA	0 (0–0), *4*	0 (0–0), *1*
Centrilobular cysts	1 (3.3%)	0	NA	0 (0–0), *2*	0 (0–0), *0*
Paraseptal cysts	2 (6.7%)	0	NA	0 (0–0), *1*	0 (0–0), *0*
Fissure thickening and distortions	8 (26.7%)	1 (12.5%)	0.65	0 (0–1), *8*	0 (0–0), *1*
Bronchiectasis	5 (16.7%)	3 (37.5%)	0.33	0 (0–0), *2*	0 (0–3), *5*
Mosaic perfusion	7 (23.3%)	0	0.31	0 (0–0), *28*	0 (0–0), *0*

*ep-ARDS* extra-pulmonary ARDS. *p-ARDS* pulmonary ARDS. Left panel: % of children with abnormality. Right panel: medians of sum of quotations (interquartile ranges), max

**Fig. 2:**
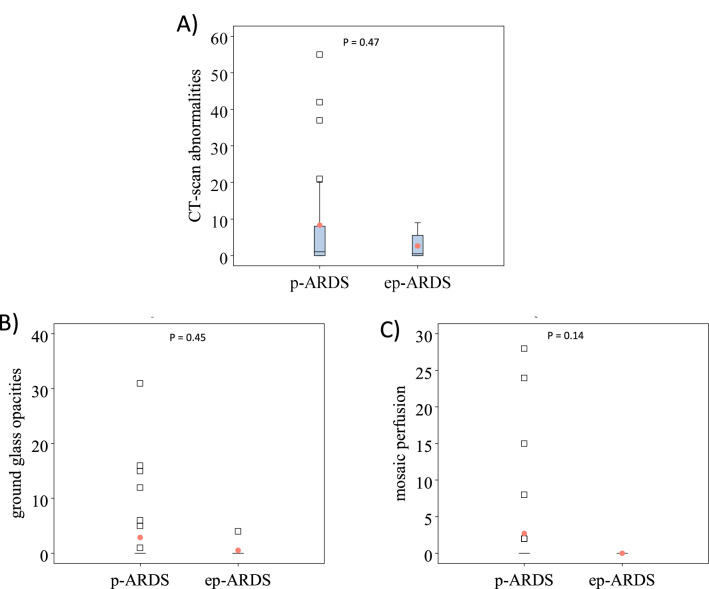
**A** Box plot describing scores of CT-scan abnormalities (sum of quotations), **B** scores of ground glass opacities and **C** scores of mosaic perfusion in pulmonary (p-ARDS) and extra-pulmonary ARDS (ep-ARDS)

The most extensive abnormalities were ground glass opacities (maximal sum of quotations reaching 31/36, Table 4) and mosaic perfusion pattern (maximal sum of quotations reaching 28/36, Table 3), with high scores observed in 5 children with p-ARDS (Fig. 2b). The ground glass opacities pattern was scored at 31, 15 and 16 in 3 patients, all with p-ARDS. The mosaic perfusion pattern was scored at 24, 28 and 15 in 3 patients, all with p-ARDS (Fig. 2c).

### PFT

PFT were obtained in 35/39 children (90%, PFT were not performed in 4 children less than 2 years of age) with FRC measurements being performed in 30/39 patients. Abnormalities in PFT were observed in 30/35 of them (86% of children) with a similar proportion of children with p-ARDS and ep-ARDS (Table 5).

**Table 5:** Summary of abnormalities in PFT (all ages) in 35 children by type of ARDS

Abnormality	*n*	p-ARDS *n* = 27 23 children (85%)	ep-ARDS *n* = 8 7 children (88%)
Hyperinflation and/or obstructive pattern	12/35	9	3
Hyperinflation [functional residual capacity (FRC), total lung capacity (TLC)]	7/30	4	3
Obstruction [resistance by interruption technique (R_int)_, total lung resistance by oesophageal-balloon technique (R_L_)]	11/19	9	2
Associated to a decrease in lung dynamic compliance (C_Ldyn_ /FRC)	7/9	7	0
Restrictive abnormalities	6/12	4	2
True restrictive ventilatory defect (TLC) in 4/11 children		3	1
and/or decrease in FVC and FEV_1_ together with a FEV_1_/FVC ratio ≥ LLN suggestive of a restrictive ventilatory defect in 4/12 children		2	2
Decrease in carbon monoxide transfer factor (T_LCO_)	3/8	2	1
Hypoxemia	2/14	1	1
Decrease in inspiratory muscle strength (SNIP)	2/13	2	0
Decrease in 6-min-walking distance	11/15	9	2
Desaturation at the end of a 6-min-walk test	1/16	1	0

*FEV*_*1*_ forced expiratory volume in 1 s, *FVC* forced vital capacity, *ep-ARDS* extra-pulmonary ARDS. *p-ARDS* pulmonary ARDS, *PFT *pulmonary function tests, *SNIP* sniff nasal inspiratory pressure

A lung hyperinflation and/or an obstructive pattern was observed in 12/35 children (34%, 9 children with p-ARDS): a lung hyperinflation in 7/30 children, and an obstructive pattern (as indicated by the increased R_int_ measurement and/or R_L_ measurements or significant decrease in these indices after bronchodilator) in 11/19 children (9 with p-ARDS). In addition, 6 children among those with a lung hyperinflation and/or an obstructive pattern (all p-ARDS) showed a decrease in C_Ldyn_.

Restrictive abnormalities were observed in 6 children of whom 4 with p-ARDS: a reduced TLC (< 80% predicted and indicating true restriction) was observed in 4 children and an isolated decrease in FVC < LLN together with an FEV_1_/FVC ratio ≥ LLN (suggestive of a restrictive ventilatory defect) was observed in 2 additional children. A mild decrease in T_LCO_ was observed in 2 children with restrictive abnormalities (transfer coefficient of the lung for carbon monoxide was within limits of normal values in these children), and a large decrease in inspiratory muscle strength was observed in one child with a concomitant decrease in FVC, suggestive of a restrictive ventilatory defect.

A decrease in the 6-min-walking distance was observed in 11/15 children (9 children with p-ARDS) including 3 children with restrictive abnormalities (including the child with a large decrease in inspiratory muscle strength) and 1 child with a lung hyperinflation and/or an obstructive pattern. In 1 child with p-ARDS, desaturation at the end of the 6-min-walk test was observed.

PFT results are detailed in Additional file 1: Table S1 for patients < 7 years of age and in Additional file 1: Table S2 for the older patients, as the tests used were different.

C_Ldyn_ was obtained in 9 children. Lung dynamic compliance was not assessed in 26 children (this invasive technique was refused or contra indicated in 13 children, and reason for not performing the measurement was not reported for 13 children additional children).

Among 28 children younger than 7 years of age (range 1.1–6.9 years), FRC was obtained in 19 children, R_int_ in 12 children, R_L_ together with C_Ldyn_ in 9 children, SNIP in 3 children (the youngest patient performing SNIP was 6.4 years old), SpO_2_ in 8 children and capillary blood gases in 11 children.

Among 11 children ≥ 7 years of age (range 7.7–16.2), FRC was obtained in 11 children with TLC in 10 children, spirometry in 11 children, SNIP in 10 children, T_LCO_ in 8 children, SpO_2_ in 8 children, arterialized capillary blood gases in 3 children and 6MWT in 8 children.

Relationship between physiologic parameters/ventilator settings collected in the PICU and CT scan, and PFTs at the 1-year assessment are presented in detail in Table 6: increasing driving pressure (max plateau pressure (Pplat)—max PEEP) was correlated with increasing CT-scan abnormalities, ground glass opacities, parenchymal consolidations and functional residual capacity (more hyperinflation), with all *p* < 0.005. Higher oxygenation index was correlated with higher R_int_, and lower C_Ldyn_. Higher driving pressure, higher maximal Pplat, higher maximal VTE*kg^−1^ and higher maximal ventilation index all increased with the degree of hyperinflation.

**Table 6:** Correlation matrix (spearman correlation) of physiologic parameters/ventilator settings, CT scan, and PFTs

	Worse PaO_2_/FiO_2_ ratio	Max OI	Max DP	Max Pplat	maxVTE × kg^−1^	Max PEEP	Max VI
CT-scan abnormalities	− 0.2254	0.23702	0.42332	0.27985	− 0.18168	0.14739	0.1027
	0.1798	0.2648	0.0278	0.1574	0.2889	0.3772	0.6252
	37	24	27	27	36	38	25
Ground glass opacities	− 0.28551	0.29183	0.41003	0.2727	− 0.08419	0.22173	0.1993
	0.0867	0.1664	0.0337	0.1688	0.6254	0.1809	0.3395
	37	24	27	27	36	38	25
Parenchymal consolidations	− 0.27678	− 0.2602	0.3842	0.15842	− 0.05876	− 0.10985	− 0.05518
	0.0972	0.2195	0.0479	0.43	0.7335	0.5115	0.7933
	37	24	27	27	36	38	25
Septal reticulation	0.03512	0.06435	− 0.09486	0.04296	− 0.10981	− 0.15434	− 0.2817
	0.8365	0.7652	0.6379	0.8315	0.5238	0.3549	0.1725
	37	24	27	27	36	38	25
Honey combing	− 0.00393	− 0.12242	0.05054	0.06321	− 0.16435	− 0.18822	− 0.08341
	0.9816	0.5688	0.8023	0.7541	0.3382	0.2578	0.6918
	37	24	27	27	36	38	25
Centrilobular cysts	− 0.26548				− 0.2034	− 0.06868	
	0.1123				0.2341	0.682	
	37	24	27	27	36	38	25
Paraseptal cysts	− 0.17358				− 0.2034	− 0.09846	
	0.3042				0.2341	0.5564	
	37	24	27	27	36	38	25
Fissure thickening and distortions	− 0.08342	− 0.11809	0.02315	− 0.22449	− 0.30531	− 0.02586	0
	0.6235	0.5826	0.9087	0.2603	0.0702	0.8775	1
	37	24	27	27	36	38	25
Bronchiectasis	− 0.05191	0.06128	0.32053	0.33418	0.11382	0.01895	0.09159
	0.7603	0.7761	0.1031	0.0884	0.5086	0.9101	0.6633
	37	24	27	27	36	38	25
Mosaic perfusion	− 0.17837	0.17357	0.14341	0.06746	− 0.29036	0.31527	0.31858
	0.2909	0.4173	0.4754	0.7381	0.0858	0.0539	0.1206
	37	24	27	27	36	38	25
FRC, % predicted	− 0.29732	0.27218	0.39217	0.38531	0.40472	0.05856	0.48912
	0.1106	0.2457	0.0475	0.0472	0.0265	0.7586	0.0244
	30	20	26	27	30	30	21
R_int_, % predicted	− 0.37828	0.83333	− 0.02462	0.1549	0.36364	0.21751	0.18333
	0.2253	0.0053	0.9462	0.6493	0.2453	0.4971	0.6368
	12	9	10	11	12	12	9
R_L_, % of predicted	− 0.05858	0.64672			0.45238	− 0.08513	− 0.41917
	0.881	0.0831	5	5	0.2604	0.8276	0.3013
	9	8			8	9	8
C_Ldyn_, % predicted	0.56067	− 0.7545			0.38095	− 0.70654	0.38324
	0.1163	0.0305	5	5	0.3518	0.0333	0.3487
	9	8			8	9	8
FVC, *z*-score	− 0.34266		− 0.53522	− 0.47101	0.37762	0.08669	6
	0.2756	5	0.0729	0.1222	0.2262	0.7888	
	12		12	12	12	12	
FEV_1_, *z*-score	− 0.25874		− 0.26409	− 0.25659	0.3007	0.0903	6
	0.4168	5	0.4069	0.4208	0.3423	0.7802	
	12		12	12	12	12	
FEV_1_/FVC ratio, *z*-score	0.09091		0.52466	0.30932	− 0.24476	0.1517	
	0.7787	5	0.0799	0.3279	0.4433	0.6379	
	12		12	12	12	12	6
SpO_2_ (end of 6MWT), %	− 0.41912	7	− 0.35946	− 0.13085	− 0.04531	0.28603	7
	0.1199		0.2277	0.67	0.8726	0.3014	
	15		13	13	15	15	
T_LCO_, *z*-score	0.2381		− 0.23953	− 0.27545	0	− 0.31709	
	0.5702		0.5678	0.5091	1	0.4441	4
	8	3	8	8	8	8	

*C*_*Ldyn*_ lung dynamic compliance, *DP* driving pressure = delta (Pplat-PEEP), *FRC* functional residual capacity, *FEV*_*1*_ forced expiratory volume in 1 s, *FVC* forced vital capacity, *OI* oxygenation index, *R*_*int*_ resistance by the interrupter method, *R*_*L*_ total lung resistance, *6MWT* 6-min-walk test, *T*_*LCO*_ carbon monoxide transfer factor, *VTE* expiratory tidal volume, *VI* ventilation index

Results are Spearman’s correlation coefficients with *p*-values

Neither CT-scan abnormalities, nor PFT abnormalities were related to respiratory symptoms and/or requirement for maintenance treatment in the year after PICUD (Additional file 1: Table S3).

## Discussion

This study reports the results of pulmonary assessment performed 1 year after PICUD of a group of children survivors of ARDS, with a clear distinction being made between p-ARDS and ep-ARDS. This is the first study describing CT abnormalities of lung parenchyma in children < 17 years old, with a relatively large study population compared to the available literature on adults [13–17]. The majority of children who were mechanically ventilated for ARDS expressed respiratory symptoms and presented CT-scan and PFT abnormalities. The severity of ARDS as indicated by physiologic parameters/ventilator settings during the PICU stay was correlated with CT scan and PFTs abnormalities at the 1-year assessment. Respiratory symptoms were reported in as much as 74% of children surviving to p-ARDS, and, sequelae observed on thoracic CT scan were important in some children, all in the p-ARDS group.

Based on the oxygen deficit (oxygenation index collected at H24 after admission for ARDS in PICU) our population consisted of mild-to-severe paediatric ARDS [3, 6]. Sixty-two percent of the 37 children in whom VTE was reported were ventilated with VTE within the 5–8 ml/kg recommended VTE range, 38% of the 37 children with VTE within the 3–6 ml/kg recommended VTE range for patients with poor Crs [12] in order to tend to a lung-protective ventilation strategy. A trend to lower VTE in the patients with poorer Crs was observed in our study. Fourteen out of the 37 children (38%) were ventilated within the 9–15 ml/kg range. In this latter group, the possibility cannot be ruled out that these high volumes contributed to some degree of volutrauma. Ventilator setting in the patients of our study did not perfectly follow the recommendations for lung-protective ventilation strategy, but were in agreement with usual care mechanical ventilation practice for paediatric ARDS [18]. Indeed, maximal VTE values/kg (using actual body weight) during the whole PICU stay were slightly lower than those observed during the first 72 h of mechanical ventilation in a multicentre study assessing variability in usual care mechanical ventilation practice for paediatric ARDS [18]. Median maximal PEEP was within the recommended ranges [12] and median maximal plateau pressure (Pplat) was higher than recommended limit value (28 cmH_2_O) [12]. Mean highest PEEP in p-ARDS and ep-ARDS groups were 1 cmH_2_O higher and 4 cmH_2_O higher, respectively, than those reported in the previous study [18] and maximal PImax was ≤ 40 cmH_2_O in 42% children in our study versus 99% in that study [18].

We must underline that the number of children with ep-ARDS is small, however respiratory symptoms at rest or exercise and/or requirement for respiratory maintenance treatment during the year following the PICU hospitalization were sixfold more frequent in children with p-ARDS, than in those with ep-ARDS with more frequent wheezing episodes in the former group. Lower respiratory tract infections and wheezing episodes persisted up to a median time after PICUD of, respectively, 10 months and 12 months. Boucher et al*.* reported abnormal respiratory symptoms (cough, wheezing with or without upper respiratory infections, respiratory symptoms on exertion) in fewer (37%) of children 3 months after PICUD though the majority of children (76%) had suffered from ARDS of respiratory infectious aetiology [2]. Chakdour et al. noticed respiratory symptoms (chronic cough ± dyspnoea) in a similar percentage (68%) of children, all with p-ARDS, 3 months after discharge [19] though none had respiratory symptoms 9–12 months after discharge. It should be noted that both studies included patients with less-severe ARDS, as assessed by PALICC criteria and patients’ characteristics.

On CT scan at 1-year follow-up, in our group of ep-ARDS, all abnormalities were observed in the anterior lung parenchyma, with septal reticulations pattern in 13% of children and ground glass opacities pattern in 13% of children. Nöbauer et al*.,* in adults with primary thoracic trauma, also observed that CT scan lesions were predominantly located in the ventral zone [17]. Desai et al*.* assessing adult ARDS survivors, 6.5 months after the acute phase, underlined that the most common abnormality observed in thoracic CT scan of ARDS survivors (mostly ep-ARDS) was a reticular pattern with predilection in the anterior nondependent zone, and that its extent was related to the duration of mechanical ventilation [16]. A reticular pattern was observed by these authors in a higher proportion of patients (85%) than in our study, but the mean duration of mechanical ventilation was longer in their population [16].

Ground glass opacification in survivors is supposed to represent fine intralobular fibrosis below the resolutions limits of CT scans [16]. In another study by Desai et al*.*, in adults, the extent of reticular pattern and ground glass opacification were negatively correlated with FVC, but positively correlated with the ratio of residual volume to total lung capacity [20].

Howling et al*.* in adults ARDS reported that dilated bronchi observed during the acute phase in 68% of the analysed lobes persisted in the majority (92%) at 6-month follow-up, often (88%) accompanied by CT features of supervening pulmonary fibrosis (reticular distorting pattern) [14]. Reticular pattern were observed in less than 20% of our cohort of child ARDS survivors. Bronchiectasis was also less likely in our cohort (21%) than in adults.

In our study, CT-scan abnormalities of p-ARDS were diffuse (both anterior and posterior). This less classical diffuse distribution of abnormalities, with areas of reticular pattern and ground-glass opacification, was observed in adults after ARDS (63% of intrapulmonary origin) [15].

In 5 of our children, all with p-ARDS, the ground glass opacities pattern and the mosaic perfusion pattern were more widespread. Kim et al. in adults also observed that, although the mean extent of lesions in all patients was mild (averaging 15.3% of the total lung volume), the lesions were more extensive in the p-ARDS than the ep-ARDS group [4]. They speculated that, due to differences in lung mechanics in the early stage of ARDS and the refractory nature of p-ARDS with respect to alveolar recruitment, p-ARDS may be more vulnerable to ventilator-induced lung injury compared with ep-ARDS, leading to more important sequelae in the long-term [4].

We found a mosaic perfusion pattern in 23% of children with p-ARDS but in none of ep-ARDS cases. Decreased attenuation consistent with a mosaic pattern was observed in 13% of ARDS adults [13], and decreased attenuation due to small airway disease in 11% of ARDS patients [16]. Disease of the lung prior to ARDS may have contributed to the observed anomalies. In 2 of the previously mentioned studies 33% and 42% of patients were smokers, respectively [13, 17]. The authors underlined that pre-existing diseases of the lung, such as smoking-related emphysema or post-infection scarring, were excluded by clinical history only not by imaging modality in their study. In other studies in adults [4, 15] and the present study, patients with previous respiratory disease or neurologic injury have been excluded in an attempt to better isolate the contribution of ARDS itself to morbidity.

PFT abnormalities were observed in 86% of this cohort including a lung hyperinflation and/or an obstructive pattern, restrictive abnormalities, a mild decrease in diffusing capacity. Important abnormalities were observed in 7 children, all with p-ARDS.

Adult survivors of ARDS may have abnormalities in pulmonary function and exercise endurance which can persist for up to 5 years [21]. A decreased 6-min-walking distance was observed at 3 months; though this last improved at 1 year [22]. In our cohort, a decrease in the 6-min-walking distance was observed in 11/15 children, desaturation at the end of the 6-min-walk test was observed in 1 child and decrease in inspiratory muscle strength in 2/13 children.

Few paediatric studies report the long-term pulmonary function of ARDS survivors [19, 23, 24] and they are often of a smaller size than our study. Abnormalities in PFT were observed in a larger proportion of our cohort (86%) than in the study of Ward et al. (37% of children), 10.7 months after p- or ep-ARDS, but ARDS in their cohort was less severe (based on the PaO_2_/FiO_2_ ratio and the PICU length of stay) [23].

Contrary to our study, Ward et al*.* [23] observed some obstructive—but no restrictive abnormalities in children with p- and ep-ARDS. In Chakdour et al*.*’s study, conducted at 9–12 months after discharge, 19% of children exhibited a restrictive pattern [19]. In a smaller sample study of 7 children with ARDS, followed up for a mean duration of 5.6 years after PICUD, PFT were within normal limits except for one child with mildly reduced T_LCO_ and another with exercise-induced hypoxemia [24]. In none of the above-mentioned paediatric studies evaluating PFT was a CT scan performed.

### Strength and limitations

The strengths of our study include its prospective design, its consistent follow-up time, the inclusion of children covering a large range of ages at the 1-year assessment, its inclusion of a significant proportion of severe paediatric ARDS with greater potential for persistent pulmonary dysfunction, and its screening for thoracic CT-scan abnormalities—to our knowledge, this is the first study describing CT abnormalities of lung parenchyma in children < 17 years of age surviving ARDS. Our study also discriminates respiratory sequelae deriving from p-ARDS and ep-ARDS—it has been shown in an adult group of patients that p-ARDS were more likely to develop pulmonary fibrosis [4]. The respiratory sequelae observed in the group of children with ep-ARDS need further confirmation as the number of children in this group was small.

Limitations of our study are that PFT outcome measures differed in older and younger children, since PFT assessment was adapted to developmental ability and cooperation (for example, in children less than 7 years of age, tests requiring no active cooperation were used in 2.5–6-year-old children or were performed during sleep in less than 2.5 years of age patients), and that dynamic lung compliance was not assessed in all children.

*In conclusion,* children surviving ARDS requiring mechanical ventilation present frequent respiratory symptoms, significant CT-scan and PFT abnormalities 1 year after PICUD. This strongly highlights the need for a systematic pulmonary assessment at 1 year of these children.

## Supplementary Information

**Additional file 1.** Methods, respiratory symtom questionnaire: medical history taken from the patient and/or his/her parents the day of the one-year respiratory assessment and items noted and collected as categorical variables.

## Data Availability

After publication, data will be made available to other investigators on reasonable requests to the corresponding author. A proposal with a detailed description of study objectives and statistical analysis plan will be needed for evaluation of the reasonability of requests. Additional materials might also be required during the process of evaluation. Deidentified participant data will be provided after approval from the corresponding author.

## Abbreviations

AECC: American European Consensus Conference
ARDS: Acute respiratory distress syndrome
C_Ldyn_: Dynamic lung compliance
Crs: Respiratory system compliance
CT: Computed tomography
DP: Driving pressure = Pplat-PEEP
ep-ARDS: Extra-pulmonary ARDS
FEV_1_: Forced expiratory volume in 1 s
FRC: Functional residual capacity
FVC: Forced vital capacity
IQR: Interquartile ranges
GLI: Global lung initiative
LLN: Lower limit of normal
PALICC: Paediatric Acute Lung Injury Consensus Conference
p-ARDS: Pulmonary ARDS
PEEP: Positive end-expiratory pressure
PFT: Pulmonary function tests
PICU: Paediatric intensive care unit
PICUD: PICU discharge
PImax: Maximal inspiratory pressure
Pplat: Plateau pressure
R_int_: Airway resistance, interruption method
6MWT: 6-Minute-walk test
SNIP: Sniff nasal inspiratory pressure measurement
TLC: Total lung capacity
T_LCO_: Diffusing capacity of the lung for carbon monoxide
VTE: Expiratory tidal volume

## References

[1] Quasney MW, López-Fernández YM, Santschi M, Watson RS, Pediatric Acute Lung Injury Consensus Conference Group (2015). The outcomes of children with pediatric acute respiratory distress syndrome: proceedings from the Pediatric Acute Lung Injury Consensus Conference. Pediatr Crit Care Med.

[2] Boucher V, Mathy C, Lacroix J, Émériaud G, Jouvet P, Tse SM (2020). Post-discharge respiratory outcomes of children with acute respiratory distress syndrome. Pediatr Pulmonol.

[3] Cheifetz IM (2017). Pediatric ARDS. Respir Care.

[4] Kim SJ, Oh BJ, Lee JS, Lim CM, Shim TS, Lee SD (2004). Recovery from lung injury in survivors of acute respiratory distress syndrome: difference between pulmonary and extrapulmonary subtypes. Intensive Care Med.

[5] Bernard GR, Artigas A, Brigham KL, Carlet J, Falke K, Hudson L, The American-European Consensus Conference on ARDS (1994). Definitions, mechanisms, relevant outcomes, and clinical trial coordination. Am J Respir Crit Care Med.

[6] Pediatric Acute Lung Injury Consensus Conference Group (2015). Pediatric acute respiratory distress syndrome: consensus recommendations from the Pediatric Acute Lung Injury Consensus Conference. Pediatr Crit Care Med.

[7] Quanjer PH, Stanojevic S, Cole TJ, Baur X, Hall GL, Culver BH (2012). Multi-ethnic reference values for spirometry for the 3–95-yr age range: the global lung function 2012 equations. Eur Respir J.

[8] Stanojevic S, Graham BL, Cooper BG, Thompson BR, Carter KW, Francis RW, et al. Official ERS technical standards: global Lung Function Initiative reference values for the carbon monoxide transfer factor for Caucasians. Eur Respir J. 2017;50(3). 10.1183/13993003.00010-2017 28893868

[9] Quanjer PH, Stanojevic S, Cole TJ, Stocks J. GLI-2012 Data Conversion software. http://www.lungfunction.org/files/Install-GLI2012_DataConversion.EXE.

[10] Quanjer PH, Tammeling GJ, Cotes JE, Pedersen OF, Peslin R, Yernault JC (1993). Lung volumes and forced ventilatory flows. Eur Respir J Suppl.

[11] Pellegrino R, Viegi G, Brusasco V, Crapo RO, Burgos F, Casaburi R (2005). Interpretative strategies for lung function tests. Eur Respir J.

[12] Rimensberger PC, Cheifetz IM, Group for the PALICC (2015). Ventilatory support in children with pediatric acute respiratory distress syndrome: proceedings from the pediatric acute lung injury consensus conference. Pediatr Crit Care Med.

[13] Wilcox ME, Patsios D, Murphy G, Kudlow P, Paul N, Tansey CM (2013). Radiologic outcomes at 5 years after severe ARDS. Chest.

[14] Howling SJ, Evans TW, Hansell DM (1998). The significance of bronchial dilatation on CT in patients with adult respiratory distress syndrome. Clin Radiol.

[15] Masclans JR, Roca O, Muñoz X, Pallisa E, Torres F, Rello J (2011). Quality of life, pulmonary function, and tomographic scan abnormalities after ARDS. Chest.

[16] Desai SR, Wells AU, Rubens MB, Evans TW, Hansell DM (1999). Acute respiratory distress syndrome: CT abnormalities at long-term follow-up. Radiology.

[17] Nöbauer-Huhmann IM, Eibenberger K, Schaefer-Prokop C, Steltzer H, Schlick W, Strasser K (2001). Changes in lung parenchyma after acute respiratory distress syndrome (ARDS): assessment with high-resolution computed tomography. Eur Radiol.

[18] Newth CJL, Sward KA, Khemani RG, Page K, Meert KL, Carcillo JA (2017). Variability in usual care mechanical ventilation for pediatric Acute Respiratory Distress Syndrome: time for a decision support protocol?. Pediatr Crit Care Med.

[19] Chakdour S, Vaidya PC, Angurana SK, Muralidharan J, Singh M, Singhi SC (2018). Pulmonary functions in children ventilated for acute hypoxemic respiratory failure. Pediatr Crit Care Med.

[20] Desai SR (2002). Acute respiratory distress syndrome: imaging of the injured lung. Clin Radiol.

[21] Herridge MS, Tansey CM, Matté A, Tomlinson G, Diaz-Granados N, Cooper A (2011). Functional disability 5 years after acute respiratory distress syndrome. N Engl J Med.

[22] Herridge MS, Cheung AM, Tansey CM, Matte-Martyn A, Diaz-Granados N, Al-Saidi F (2003). One-year outcomes in survivors of the acute respiratory distress syndrome. N Engl J Med.

[23] Ward SL, Turpin A, Spicer AC, Treadwell MJ, Church GD, Flori HR (2017). Long term pulmonary function and quality of life in children after acute respiratory distress syndrome: a feasibility investigation. Pediatr Crit Care Med.

[24] Ben-Abraham R, Weinbroum AA, Roizin H, Efrati O, Augarten A, Harel R (2002). Long-term assessment of pulmonary function tests in pediatric survivors of acute respiratory distress syndrome. Med Sci Monit.

