# Supplementary material for: Whole pulmonary assessment 1 year after paediatric acute respiratory distress syndrome: prospective multicentre study
Source: Ann Intensive Care. 2022 Aug 20;12:79. doi: 10.1186/s13613-022-01050-4 (PMC9392829; doi:10.1186/s13613-022-01050-4)
Supplement: Supplementary file 1 — Additional file 1. Methods, respiratory symtom questionnaire: medical history taken from the patient and/or his/her parents the day of the one-year respiratory assessment and items noted and collected as categorical variables. [file 13613_2022_1050_MOESM1_ESM.doc]

**Whole pulmonary assessment 1 year after paediatric acute respiratory distress syndrome: prospective multicentre study**

Véronique Nève^1,2,3^, Ahmed Sadik^4^, Laurent Petyt^5^, Stéphane Dauger^6^, Ahmed Kheniche^7^, André Denjean^8^, Pierre-Louis Léger^9^, François Chalard^10^, Michèle Boulé^11^, Etienne Javouhey^12^, Philippe Reix^13^, Isabelle Canterino^14^, Valérie Deken^15,16^, Régis Matran^1,2,3^, Stéphane Leteurtre^4,16^, Francis Leclerc^4,16^.

**Online supplement**

**Abbreviations:** C_Ldyn_ = dynamic lung compliance; ep-ARDS = extra pulmonary ARDS; FEF_25-75 =_ mean forced expiratory flow between 25% and 75% of the FVC; FEV_1_ = forced expiratory volume in 1 sec; FRC = functional residual capacity; FVC = forced vital capacity; IQR = interquartile ranges; K_CO_ index = transfer coefficient of the lung for carbon monoxide; NA = Not applicable; p-ARDS = pulmonary ARDS; PFT = pulmonary function tests; PICU = paediatric intensive care unit; PICUD = PICU discharge; R_int_ = Resistance by interruption; R_L_ = lung resistance by oesophageal-balloon technique; RV= residual volume; SEE= standard error of the estimate. SNIP = sniff nasal inspiratory pressure; TLC = total lung capacity; T_LCO =_ diffusing capacity of the lung for carbon monoxide. 6MWT = 6-minute-walk test; ΔR_int_ after bronchodilator (BD) = (after bronchodilator absolute value of R_int_ –before bronchodilator absolute value of R_int_)/predicted value of R_int_)

**Methods**

**Respiratory symptom questionnaire**

A detailed medical history was taken from the patient and/or his/her parents the day of the one-year respiratory assessment. The following items were noted and collected as categorical variables (yes/no):

-chronic cough (*i.e.* having lasted at least 2 months in the previous year), whether dry or productive, and its timing (night-time cough, cough following exercise or laughing …),

-wheezing episodes (number of wheezing episodes since paediatric intensive care unit discharge (PICUD) and date of the last one),

-episodes of lower respiratory tract infection (bronchitis, pneumonia, number of episodes since PICUD and date of the last one),

-respiratory symptoms during daily activities or sport activities: dyspnoea during daily activities (*i.e.* while walking or climbing one step stairs) or dyspnoea while exercising (*i.e.* during sport activities, running), cough or wheezing,

-respiratory treatment during the year preceding the one-year assessment, including inhaled treatments, general corticosteroids administered for lower respiratory tract symptoms.

**RESULTS**

**Pulmonary function tests (PFT)**

**Table S1** Pulmonary function tests in children < 7 years of age (n = 28*)

|  | n | values | Comments |
| --- | --- | --- | --- |
| Age, years | 28 | 2.8, 2 (1.4 to 3.9) |  |
| Height, cm | 22 | 93.1, 92 (80 to 103) |  |
| Weight, kg | 22 | 14, 13.5 (10 to 16.4) |  |
| FRC, L | 19 | 0.55, 0.47 (0.39 to 0.76) |  |
| FRC, % predicted | 19 | 122, 110.9 (97.2 to 139.4) | 6 Pts with FRC > 120% predicted, indicating lung hyperinflation and 1 Pt with restrictive ventilatory defect**^†^** |
| Baseline R_int_, kPa.L^-1^.s | 12 | 1.42, 1.37 (0.83 to 1.97) |  |
| Baseline R_int_, % predicted | 12 | 154.2, 145.2 (100 to 201.4) | R_int_ was increased in 5/12 Pts |
| ΔR_int_ after BD, % predicted | 6 | -39.3, -46.7 (-59.7 to -15.9) | R_int_ measurement obtained in 6 Pts after BD, with significant (-35% of predicted value) decrease in 4 Pts, and no significant decrease in 2 Pts. |
| C_Ldyn_, ml/cmH_2_O | 9 | 18.7, 19.5 (18 to 20) |  |
| C_Ldyn_, % predicted | 9 | 83.8, 94.8 (65.7 to 100.6) | 4 Pts with C_Ldyn_ < 75% predicted. |
| C_Ldyn_/FRC, % predicted | 7 | 65.2, 63.9 (56.6 to 74.4) | 6 Pts with (C_Ldyn_ /FRC) < 75% predicted. |
| R_L_, kPa.L^-1^.s | 9 | 32.4, 31.9 (15.6 to 40.9) |  |
| R_L_, % predicted | 9 | 250, 231.6 (162.9 to 264.8) | 5 Pts < 3 years of age with R_L_ > 200% predicted |
| SNIP, cmH_2_O | 3 | 79, 79 (78 to 80) |  |
| SNIP, z-score | 3 | -0.54, -0.57 (-0.60 to -0.46) |  |
| SNIP, % predicted | 3 | 85.7, 85.1 (84.3 to 87.7) |  |
| PaO_2_, mmHg | 11 | 82.7, 84 (75 to 93) | Hypoxemia (PaO_2_ < 2 standard deviations) in 2 children |
| PaCO_2_, mmHg | 11 | 36.6, 36 (33 to 42) |  |
| pH | 11 | 7.39, 7.39 (7.38 to 7.43) |  |
| SpO_2_ at rest, % | 8 | 99, 99 (98.5 to 99.5) |  |
| SpO_2_ at end of 6-min-walk test, % | 7 | 97.7, 97 (97 to 99) | At the end of the 6-min-walk test, no child exhibited desaturation |
| Distance walked, m | 7 | 389.7, 370 (336 to 431) |  |
| Distance walked, % predicted | 7 | 78.1, 79.9 (59.9 to 87.9) | 3 children walked a distance < lower limit of normal  (< -1.645 x SEE). |

*No pulmonary function tests (PFT) in 4 patients (Pts), only capillary blood gases in 3 additional Pts. **^†^**One Pt, aged 6.82 years, performed forced and slow spirometry that a showed restrictive ventilatory defect (total lung capacity < 80% of predicted).

C_Ldyn_ = lung dynamic compliance by the oesophageal catheter technique. FRC = functional residual capacity (helium dilution technique). R_int =_ resistance measurement by the interruption method. R_L_ = total lung resistance by the oesophageal catheter technique. ΔR_int_ after bronchodilator (BD) = (after bronchodilator absolute value of R_int_ –before bronchodilator absolute value of R_int_)/predicted value of R_int_). SNIP = sniff nasal inspiratory pressure measurement. SEE = standard error of the estimate.

Results are means, medians (interquartile ranges (IQR)).

**Table S2** Pulmonary function tests in children ≥ 7 years of age (N=11)

|  | N | Values | Comments |
| --- | --- | --- | --- |
| Age, years | 11 | 11.5, 11 (9.3 to 14.2) |  |
| Height, cm | 11 | 143.4, 144 (131 to 155) |  |
| Weight, kg | 11 | 40, 42 (28 to 45.9) |  |
| FRC, L | 11 | 1.22, 1.16 (1.12 to 1.49) |  |
| FRC, % predicted | 11 | 74.2, 73.3 (61.4 to 91.2) | 8 Pts with FRC < 80% predicted |
| TLC, L | 10 | 2.99, 2.99 (2.75 to 3.32) |  |
| TLC, % predicted | 10 | 97.1, 97.5 (78.7 to 115) | 3 Pts with restrictive ventilatory defect (*i.e.* TLC < 80% predicted) and 1 Pt with hyperinflation (*i.e.* TLC > 120% predicted) |
| VR/CPT | 10 | 0.25, 0.25 (0.21 to 0.26) |  |
| FVC, L | 11 | 2.16, 2.14 (1.69 to 2.63) |  |
| FVC, z-score | 11 | -1.06, -1.32 (-1.78 to -0.08) | FVC < -1.64 z-score in 4 Pts with FEV_1_/FVC ratio ≥ -1.64 z-score |
| FEV_1_, L | 11 | 1.87, 1.89 (1.51 to 2.22) |  |
| FEV_1_, z-score | 11 | -1.05, -1.32 (-1.77 to -0.28) | FEV_1_ < -1.64 z-score in 4 Pts |
| FEV_1_/FVC ratio | 11 | 0.87, 0.87 (0.84 to 0.89) |  |
| FEV_1_/FVC, z-score | 11 | -0.04, -0.38 (-0.71 to 0.20) | No obstructive ventilatory defect |
| FEF_25-75_, L/s | 11 | 2.36, 2.14 (1.98 to 2.90) |  |
| FEF_25-75_, z-score | 11 | -0.45, -0.77 (-0.99 to 0.19) |  |
| SNIP cmH_2_O | 10 | 85.3, 85 (64 to 117) |  |
| SNIP, z-score | 10 | -0.72, -0.72 (-1.33 to 0.09) | SNIP < -1.64 in 2 Pts |
| SNIP, % predicted | 10 | 84, 83.6 (67.2 to 102.2) |  |
| FVC after salbutamol, L | 10 | 2.16, 2.15 (1.71 to 2.71) |  |
| ΔFVC % of baseline | 10 | -1.6, -0.5 (-5.9 to 3) | No significant increase in FVC |
| FEV_1_ after salbutamol, L | 10 | 1.88, 1.94 (1.60 to 2.28) |  |
| ΔFEV_1_% of baseline | 9 | 0.7, 2.1 (0.8 to 3.5) | No significant increase in FEV_1_ |
| FEF_25-75_ after salbutamol, L/s | 10 | 2.64, 2.65 (2.32 to 2.88) |  |
| ΔFEF_25-75_ % of baseline | 9 | 12.4, 13.5 (6.7 to 17.9) | Significant increase in FEF_25-75_ in 1 Pt |
| T_LCO_ single breath, mL∙min^-1^∙mmHg^-1^ | 8 | 17.2, 16 (13.2 to 21.1) |  |
| T_LCO_ single breath, % predicted | 8 | 91.8, 85.8 (74.7 to 101.7) |  |
| T_LCO_ single breath, z-score | 8 | -0.64, -0.91 (-1.83 to 0.16) | Decreased in 2 Pts |
| K_CO_ single breath, z-score | 8 | 0.17, -0.63 (-1.36 to 1.28) | Decreased in 1 Pt |
| PaO_2_, mmHg | 3 | 93, 95 (85 to 99) |  |
| PaCO_2_, mmHg | 3 | 37.3, 38 (33 to 41) |  |
| pH | 3 | 7.45, 7.43 (7.41 to 7.51) |  |
| SpO_2_ rest, % | 8 | 98.5, 99 (98 to 99) |  |
| SpO_2_ (end of 6-min-walk test), % predicted | 8 | 96.9, 97 (96.5 to 97.5) | Desaturation < 96% in 1 Pt |
| Distance walked (6-min-walk test), m | 8 | 444.5, 431.5 (422 to 473.5) |  |
| 6-min-walking distance, % predicted | 8 | 68.3, 63.6 (62.8 to 77.2) | 8 Pts walked a distance < -1.645 x SEE |

FEF_25-75_ = mean forced expiratory flow between 25% and 75% of the FVC. FEV_1_ = forced expiratory volume in 1 s. FRC = functional residual capacity (helium dilution technique). FVC = forced vital capacity. SEE = standard error of the estimate. TLC = total lung capacity. TLCO = carbon monoxide transfer factor (single breath).

Results are means, medians (interquartile ranges (IQR).

**Agreement between PFT and thoracic CT scan abnormalities**

Neither CT scan abnormalities, nor PFT abnormalities were related to respiratory symptoms and/or requirement for maintenance treatment within the year after PICUD (Table S3).

**Table S3** Relationship between respiratory symptoms and CT scan abnormalities and PFT abnormalities

| Respiratory symptoms in the year after PICU discharge | No  N=16 | Yes  N=23 | χ^2^, p value |
| --- | --- | --- | --- |
| CT scan abnormality in 22/38 children | 8/15 (53.3%) | 14/23 (60.9%) | 0.64 |
| PFT abnormality in 30/35 children | 11/14 (78.6%) | 19/21 (90.5%) | NA |
| with decreased C_Ldyn_ in 7/9 children | 3/5 (60.0%) | 4/4 (100.0%) | NA |

C_Ldyn_: lung dynamic compliance. NA: not applicable.

**References**

1. Quanjer PH, Stanojevic S, Cole TJ, Baur X, Hall GL, Culver BH, Enright PL, Hankinson JL, Ip MS, Zheng J, et al. Multi-ethnic reference values for spirometry for the 3-95-yr age range: the global lung function 2012 equations. Eur. Respir. J. 2012; 40: 1324–1343.

2. Stanojevic S, Graham BL, Cooper BG, Thompson BR, Carter KW, Francis RW, Hall GL, Global Lung Function Initiative TLCO working group, Global Lung Function Initiative (GLI) TLCO. Official ERS technical standards: Global Lung Function Initiative reference values for the carbon monoxide transfer factor for Caucasians. Eur. Respir. J. 2017; 50.

3. Quanjer PH, Stanojevic S, Cole TJ, Stocks J. GLI-2012 Data Conversion software. http://www.lungfunction.org/files/Install- GLI2012_DataConversion.EXE. .

4. Quanjer PH, Tammeling GJ, Cotes JE, Pedersen OF, Peslin R, Yernault JC. Lung volumes and forced ventilatory flows. Report Working Party Standardization of Lung Function Tests, European Community for Steel and Coal. Official Statement of the European Respiratory Society. Eur Respir J Suppl 1993; 16: 5–40.

5. Pellegrino R, Viegi G, Brusasco V, Crapo RO, Burgos F, Casaburi R, Coates A, van der Grinten CPM, Gustafsson P, Hankinson J, et al. Interpretative strategies for lung function tests. Eur. Respir. J. 2005; 26: 948–968.
